# Quantification of thonningianin a in rat plasma by liquid chromatography tandem mass spectrometry and its application to a pharmacokinetic study

**DOI:** 10.1080/13880209.2021.1913188

**Published:** 2021-04-29

**Authors:** Qian Liu, Chunmin Li, Pan Zhao, Jing Li, Zhipeng Deng

**Affiliations:** a College of Pharmacy, Shandong University of Traditional Chinese Medicine, Jinan, PR China; b Department of Pharmacy, Jinan Maternity and Child Care Hospital, Jinan, PR China

**Keywords:** *Penthorum chinense*, liver diseases, LC-MS/MS, method validation

## Abstract

**Context:**

Thonningianin A is an ellagitannin substance and displays multiple pharmacological activities.

**Objective:**

This study investigated the pharmacokinetic characteristics of thonningianin A after oral administration in rats using a fully validated liquid chromatography tandem mass spectrometry (LC-MS/MS) method.

**Materials and methods:**

A sensitive and selective LC-MS/MS assay was developed for quantifying thonningianin A. Eighteen Wistar rats were randomly divided into three groups (*n* = 6), which were given at a single dose of 10, 20, or 40 mg/kg thonningianin A by gavage. Blood samples (200 µL) were collected from the orbit vein at designated time points and analyzed using the LC-MS/MS method to measure the levels of thonningianin A.

**Results:**

Thonningianin A and internal standard (IS) were eluted at 1.5 and ∼3.0 min, respectively. The selected reaction mode transitions monitored were *m/z* 873.2 > 300.3 and 819.3 > 610.6 for thonningianin A and the IS, respectively. The calibration range was 10–1200 ng/mL. The intra- and the inter-day accuracy and precision met the acceptance criteria. No carryover and matrix effect were observed. The plasma concentrations of thonningianin A increased rapidly after oral administration of three dosages and reached the mean peak concentrations (*C*_max_) within 0.61–0.83 h. Meanwhile, AUC_0–t_/AUC_0–∞_ of the three dosage groups was more than 89.0% (10 mg/kg), 95.7% (20 mg/kg), and 97.0% (40 mg/kg).

**Discussion and conclusions:**

The present method is the first report in terms of the simple precipitation procedure, high sensitivity, and high-throughput efficiency. This validated assay was successfully applied to determine the pharmacokinetic behaviours of thonningianin A in rats. This study should be helpful for providing references for understanding the action mechanism and further application of *Penthorum chinense*.

## Introduction

**Figure 1.**
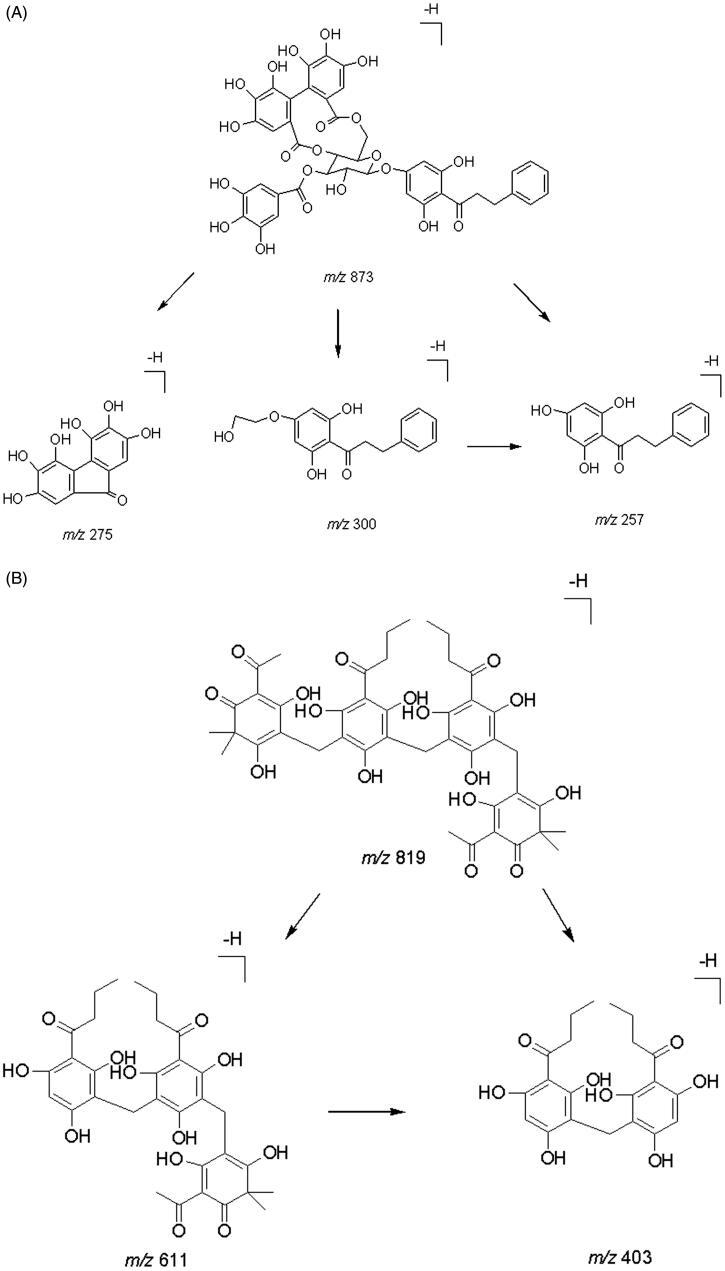
Proposed MS/MS fragmentation pathway of thonningianin A (A) and IS (B).

*Penthorum chinense* Pursh (Saxifragaceae), known as ‘Gan Huang Cao’ in Traditional Chinese Medicine, has functions in heat alleviation, diuresis, detoxification, and blood circulation promotion (Lu et al. 2012; Jeong et al. 2019). In recent years, *P. chinense* extracts have been developed into tea, functional drinks, or medicines for the treatment of several liver diseases, such as alcoholic liver disease, non-alcoholic fatty liver disease, hepatic virus infections, and liver fibrosis (Cao et al. 2015; He et al. 2019; Wang et al. 2020). Modern pharmacological studies revealed that *P. chinense* has an anti-hyperglycaemic effect on streptozotocin-induced diabetic rats and starch-induced postprandial hyperglycaemic mice (Sun et al. 2014a, 2014b; Huang et al. 2015). A recent study confirmed the prebiotic activity of *P. chinense* towards a beneficial environment for host health (Yin et al. 2020). Thonningianin A (Figure 1), an ellagitannin, is isolated from the methanolic extract of the African medicinal herb, *Thonningia sanguinea* Vahl (Balanophoraceae) (Gyamfi and Aniya 2002). Thonningianin A has antioxidant, anti-hyperglycaemic, and antibacterial properties and is a potent inhibitor of rat liver gluthathione *S*-transferase activities (Gyamfi et al. 2004; Huang et al. 2015; Ding et al. 2019). An *in vitro* experiment using serially diluted microbroth assays demonstrated that thonningianin A has anti-*Staphylococcus aureus* activity (Ding et al. 2019). In addition, thonningianin A could alleviate oxidative stress in human umbilical vein endothelial cells and the aortic artery of ApoE-KO mice; these results provide an insight into the therapeutic application of thonningianin A in atherosclerosis or other cardiovascular diseases (Sun et al. 2020).

High-performance liquid chromatography (HPLC) with UV, HPLC with mass spectrometry (MS) detection, and ultra-performance liquid chromatography with quadrupole time-of-flight methods have been established for the analysis of thonningianin A in crude herbs (Guo et al. 2015; Huang et al. 2015; Ding et al. 2019; Pompermaier et al. 2020). However, the quantification of thonningianin A in biological samples and its pharmacokinetics are not available elsewhere until now. In the present study, a sensitive and selective liquid chromatography tandem mass spectrometry (LC-MS/MS) assay was developed to quantify thonningianin A. The method was successfully applied to measure thonningianin A levels in rat pharmacokinetic studies. The authors believe that this study is the first report on the LC-MS/MS quantification and pharmacokinetics of thonningianin A.

## Materials and methods

### Chemicals and reagents

Thonningianin A (Figure 1(A), purity 99.5%) and dryocrassin ABBA (Figure 1(B), purity 99.0%, internal standard [IS]) were provided by Chengdu Herbpurify Co. Ltd. (Chengdu, China). All other HPLC-grade reagents, such as acetonitrile and methanol were obtained from Fisher Scientific (Fair Lawn, NJ). Ultrapure water was used in the experiment.

### Animals

Eighteen Wistar rats with a body weight of 220 ± 20 g were purchased from Jinan Pengyue Experimental Animal Breeding Co., Ltd. (Jinan, China) and housed in an environmentally controlled breeding area (temperature 22 ± 2 °C, relative humidity 55 ± 5%, 12 h light/dark cycle). The experimental protocol was approved by the Animal Ethics Committee of Shandong University of Traditional Chinese Medicine (Jinan, China).

### Instruments and LC-MS/MS conditions

Chromatographic analysis was performed using the Ultimate 3000 HPLC system (Dionex, Sunnyvale, CA) equipped with a temperature-controlled autosampler and a column oven. Chromatographic separation was achieved using a Syncronis™ C_18_ column (50 mm × 2.1 mm; internal diameter [i.d.], 3.0 μm; Thermo Fisher Scientific Inc., San Jose, CA). The mobile phase consisted of acetonitrile (solvent A) and water (solvent B) and ran in a gradient mode at a flow rate of 0.45 mL/min. The gradient program was set as follows: 65% of solvent A was kept constant for the first 1.0 min and then increased to 95% in 0.2 min. The percentage of solvent A was then kept at 95% until 4.5 min. Solvent A was reduced to 65% at 4.6 min and equilibrated at 65% until 6.0 min. The sample temperature in the autosampler vials was kept at 12 °C, and the column temperature was maintained at 35 °C throughout the whole analysis. Each sample (3 μL) was injected into the LC-MS/MS system for analysis.

**Figure 2.**
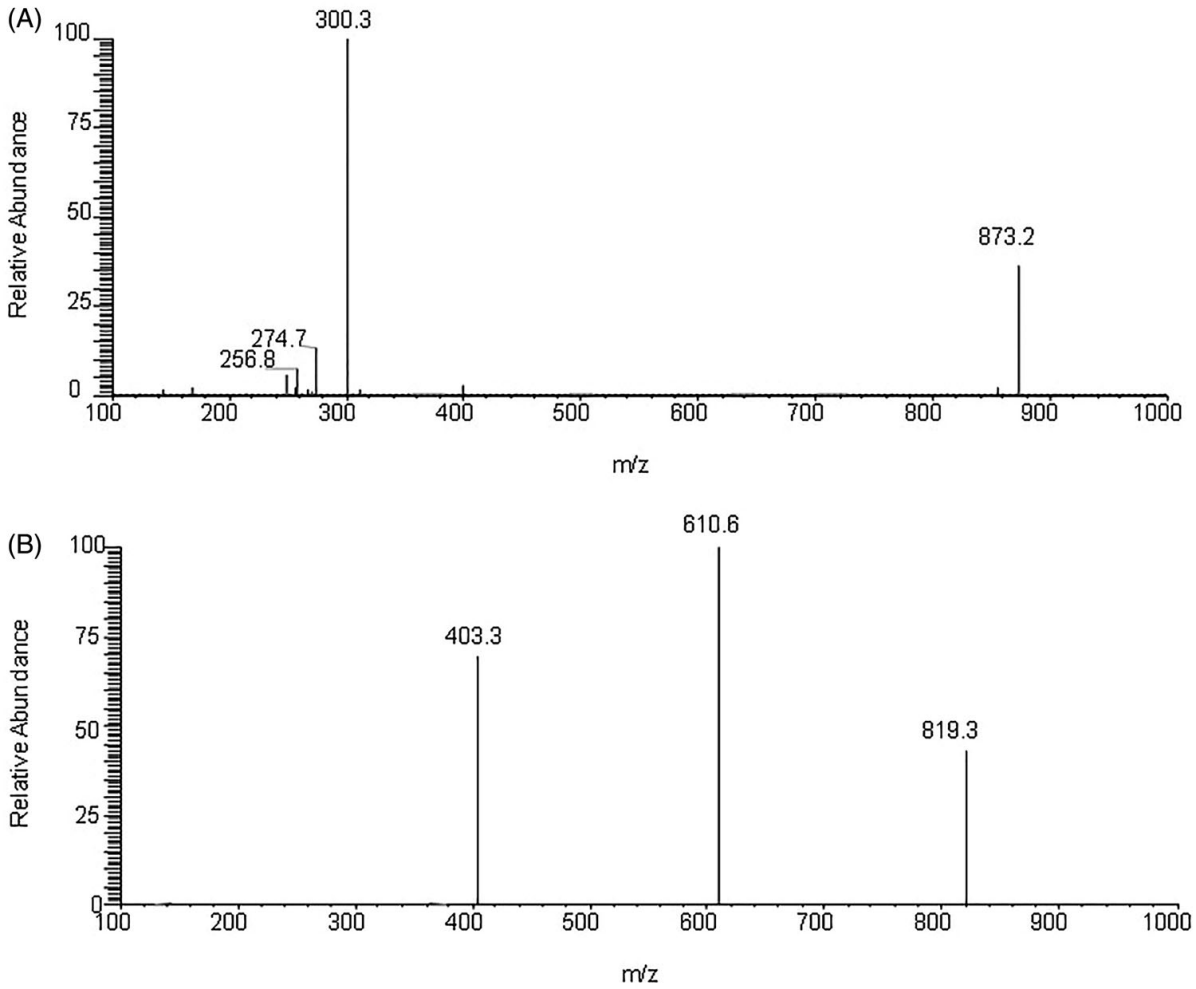
Full scan product ion spectra of thonningianin A (A) and IS (B).

**Figure 3.**
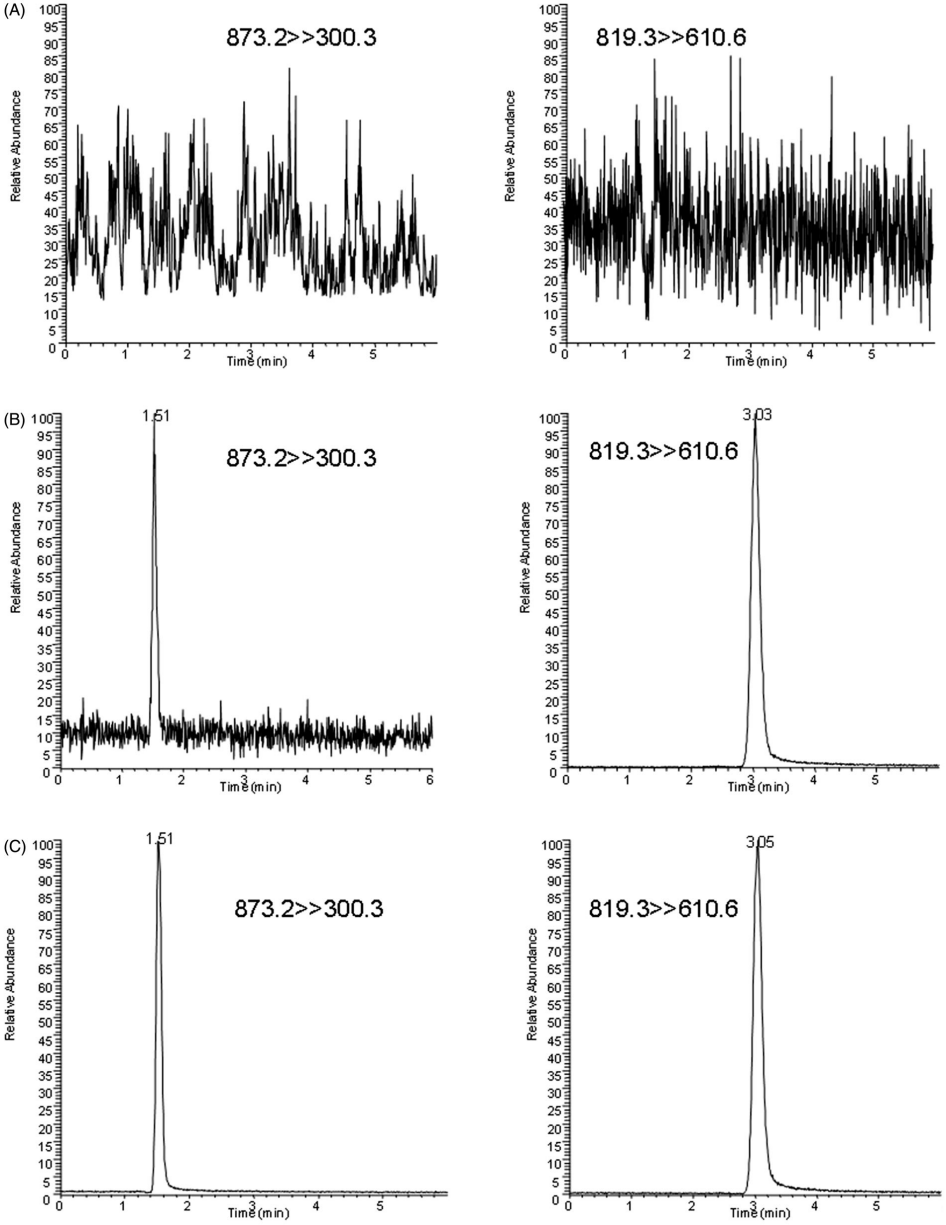
SRM chromatograms of thonningianin A and IS in a blank plasma sample (A), a blank sample spiked the analyte (LLOQ) and IS (B), and a plasma sample at 0.083 h after oral administration of 20 mg/kg thonningianin A (C).

The HPLC system was coupled with a TSQ Quantum Ultra triple quadrupole MS system (Thermo Fisher Scientific Inc., San Jose, CA) equipped with an electrospray ion source operated in negative ion mode. The MS parameters were optimized as follows: vaporizer temperature, 300 °C; capillary voltage, 3.5 kV; capillary temperature, 350 °C; sheath gas, 35 AU; and auxiliary gas, 10 AU. Thonningianin A and IS were detected using the selected-reaction mode (SRM) scan with transition ions *m/z* 873.2 > 300.3 and 819.3 > 610.6, respectively (Figures 2 and 3). The optimal collision energies of thonningianin A and the IS were 50 and 34 eV, respectively. All data were processed using the Xcalibur 2.1 software (SPSS Inc., Chicago, IL).

### Standard solution and quality control (QC) samples

Stock solutions of thonningianin A and the IS were separately prepared by dissolving the two compounds in methanol at a final concentration of 1.0 mg/mL. The thonningianin A solution was serially diluted to obtain standard working solutions (0.2, 0.8, 2, 4, 8, 16, and 24 µg/mL) and QC solutions (0.5, 2.5, and 20 µg/mL) in methanol. Calibration standard samples (10, 40, 100, 200, 400, 800, and 1200 ng/mL) were prepared by spiking 10 μL of the standard working solutions with 190 μL of blank rat plasma. QC samples at low, medium, and high concentrations (25, 125, and 1000 ng/mL, respectively) were prepared independently in the same manner. The IS working solution was prepared in methanol to yield a final concentration of 100 ng/mL.

### Sample preparation

A 50 μL aliquot of the IS working solution (200 ng/mL) and 50 μL of plasma were added into a 10-mL plastic tube with cap. The mixture was vortexed for 30 s and then added with 200 μL of acetonitrile. The mixture was extracted by vortex mixing for 5 min and centrifuged at 12,000 rpm for 10 min. The supernatant was transferred into another clean tube and evaporated to dryness with nitrogen at 30 °C. The residual was reconstituted with 100 μL of the initial mobile phase solution, and a 5 μL aliquot was injected into the LC-MS/MS system for analysis.

### Method validation

The determination method for thonningianin A level in rat plasma was validated for specificity, sensitivity, linearity, precision and accuracy, recovery, matrix effects, carryover, and stability (US Food and Drug Administration [FDA], 2018).

### Specificity

The specificity of the method was evaluated by comparing six samples of blank rat plasma with the corresponding plasma samples spiked with thonningianin A or the IS. Each sample was checked according to the preparation and analytical conditions to guarantee no interference at the retention times of thonningianin A and the IS.

### Linearity and sensitivity

The calibration curves for thonningianin A at a drug plasma concentration range of 10–1200 ng/mL were constructed for sample analysis using linear regression with a weighted factor of 1/*X*^2^, where the concentrations of thonningianin A are depicted in the *x*-axis and the peak area ratios of thonningianin A/IS are presented in the *y*-axis. The sensitivity of the method was evaluated by the lower limit of quantification (LLOQ), which is defined as the lowest concentration on the calibration curve. The criteria of precision and accuracy at the LLOQ level should not exceed ±20%.

### Precision and accuracy

The intra- and inter-day precision and accuracy of the method for thonningianin A determination were performed by analyzing five replicates of the QC samples (25, 125, and 1000 ng/mL) on the same day and on three separate days, respectively. The precision was expressed as percent relative standard deviation (%RSD), and the accuracy was calculated as the deviation of the mean from the nominal value. The acceptable criteria for accuracy was within ± 15%, and that for precision was <15% RSD.

### Extraction recovery and matrix effect

Extraction recovery was evaluated using five QC samples at each QC level by comparing the peak areas of the analytes from the post-extraction samples with those of the pre-extraction samples. Matrix effects were measured using five QC samples at each QC level by comparing the peak areas of the analytes from the post-extraction samples with those of the standard solutions.

### Carryover

Carryover was studied by evaluating the injection of blank plasma samples after the highest calibration standard. The carryover effect required less than 20% of the LLOQ for thonningianin A and 5% of the IS.

### Stability

Five QC samples at each level were checked under the following conditions to test the stability of thonningianin A in plasma undergoing different experimental conditions: 20 °C for 6 h (short-term stability); three −20 to 20 °C cycles (freeze–thaw stability); −20 °C for 40 days (long-term stability); autosampler at 12 °C for 24 h (post-preparation stability).

### Pharmacokinetic application

The validated method was applied to the pharmacokinetic study of thonningianin A in rats. All the animals were housed for 1 week before administration with free access to standard chow and water. The rats were randomly divided into three groups (*n* = 6 per group) and fasted for 12 h with free access to water before drug administration. Three groups were administered doses of 10, 20, or 40 mg/kg by gavage. Blood samples (200 µL) were collected from the orbit vein into heparinized tubes at 0 (pre-dose), 0.083, 0.17, 0.33, 0.67, 1, 2, 3, 5, 8, 12, and 24 h after oral administration. The plasma was then immediately centrifuged at 3500 rpm for 10 min to harvest plasma. Plasma samples were then frozen at −20 °C until further detection.

## Results and discussion

### Mass optimization

Negative ion electrospray mode was applied because the analyte and IS are polyphenol compounds. Thonningianin A (Figure 2(A)) gave rise to *m/z* values of 256.8, 274.7, and 300.3 from its [M–H]^−^ (*m/z* 873.2). Dryocrassin ABBA (Figure 2(B)) gave rise to major fragment ions at *m/z* 403.3 and 610.6 from its [M–H]^−^ (*m/z* 819.3). The proposed MS/MS fragmentation pathways of thonningianin A and the IS are shown in Figure 1.

### Chromatography optimization

Chromatographic conditions were optimized by examining various mobile phase and stationary phase compositions. Reversed-phase Syncronis™ C_18_ column (50 × 2.1 mm; i.d., 3.0 μm) and Hypersil™ ODS C_18_ column (50 mm × 3.0 mm; i.d., 5.0 μm) were used. Different mobile phase compositions and elution modes were examined. A gradient elution program with a mobile phase comprising of acetonitrile (solvent A) and water (solvent B) was applied to improve peak shapes and achieve good selectivity. The program had satisfactory separation efficiency. In the current study, protein precipitation procedure using acetonitrile as an extracting agent resulted in high extraction recovery and good peak shape for thonningianin A and IS.

### Method validation

#### Specificity and carryover

The representative SRM chromatograms of the blank rat plasma, blank rat plasma supplemented with thonningianin A at LLOQ and the IS, and a real plasma sample collected at 0.083 h post-drug administration are displayed in Figure 3. No endogenous interference occurred in the detection of thonningianin A and the IS. No carryover was observed in the current study.

#### Linearity and sensitivity

Good linearity was achieved over the seven concentrations of thonningianin A. The regression equation was *y* = 0.008797*x* ± 0.02341 (*r* = 0.9982). All standard deviations were less than 15% of nominal concentrations. The LLOQ was determined to be 10 ng/mL, and the precision and the accuracy fell within ± 15% (Table 1).

**Table 1.** Precision and accuracy of the LC-MS/MS method (*n* = 5).

Spiked conc. ng/mL	Intra-day			Inter-day		
	Measured conc. ng/mL	Precision (%)	Accuracy (%)	Measured conc. ng/mL	Precision (%)	Accuracy (%)
10	11.4 ± 0.4	3.8	13.5	10.6 ± 0.1	0.9	6.1
25	22.9 ± 0.3	1.4	–8.4	25.3 ± 3.4	13.5	1.1
125	122.9 ± 7.7	6.3	–1.7	121.3 ± 6.8	5.6	–3.0
1000	1098.0 ± 24.2	2.2	9.8	939.0 ± 95.8	10.2	–6.1

#### Precision and accuracy

The intra‐ and inter‐day precision and accuracy are summarized in Table 1. The intra‐ and inter‐day precision were less than 15% with a maximum RSD value of 13.5%. The intra‐ and inter‐day accuracy were ranged from −8.4% to 9.8%. These data demonstrated that the present method is reliable and reproducible for the determination of thonningianin A in rat plasma.

#### Stability

The stability of the analytes in rat plasma was determined under the following conditions: at 20 °C for 6 h, −20 °C for 40 days, at 12 °C for 24 h in the autosampler; and after three freeze-thaw cycles (± 20 °C). The results in Table 2 indicate that thonningianin A was stable in rat plasma under the tested conditions. All the accuracies (in percent relative error) ranged from −11.7% to 12.7%.

**Table 2.** Stability of the thonningianin A in rat plasma (*n* = 5).

Spiked conc. ng/mL	Stability (RE, %)			
	20 °C for 6 h	–20 °C for 40 days	Autosampler at 12 °C for 24h	Three freeze and thaw cycles
25	–8.8	12.7	–5.6	–4.1
125	5.7	9.8	–11.7	–3.8
1000	2.5	4.9	4.8	6.3

#### Extraction recovery and matrix effects

The extraction recoveries of thonningianin A were 95.8 ± 7.8%, 94.9 ± 5.8%, and 91.5 ± 5.0% at the three QC levels (25, 125, and 1000 ng/mL), respectively. The mean matrix effects were 99.0 ± 4.3%, 101.9 ± 6.2%, and 95.5 ± 2.5% for thonningianin A at the three QC concentrations, respectively (*n* = 6, Table 3). In addition, the recovery and matrix effect for the IS were 92.0 ± 3.4% and 99.4 ± 8.3% at a dose of 200 ng/mL, respectively. The results indicated that no obvious ion enhancement or suppression occurred in the plasma matrix for thonningianin A.

**Table 3.** Recovery and matrix effect of the thonningianin A and IS in rat plasma (*n* = 5).

Analyte	Spiked conc. ng/mL	Extraction recovery (%)	RSD (%)	Matrix effect (%)	RSD (%)
Thonningianin A	25	95.8 ± 7.8	8.1	99.0 ± 4.3	4.3
	125	94.9 ± 5.8	6.1	101.9 ± 6.2	6.1
	1000	91.5 ± 5.0	5.5	95.5 ± 2.5	2.6
Dryocrassin ABBA (IS)	200	92.0 ± 3.4	3.7	99.4 ± 8.3	8.4

### Pharmacokinetic study

**Figure 4.**
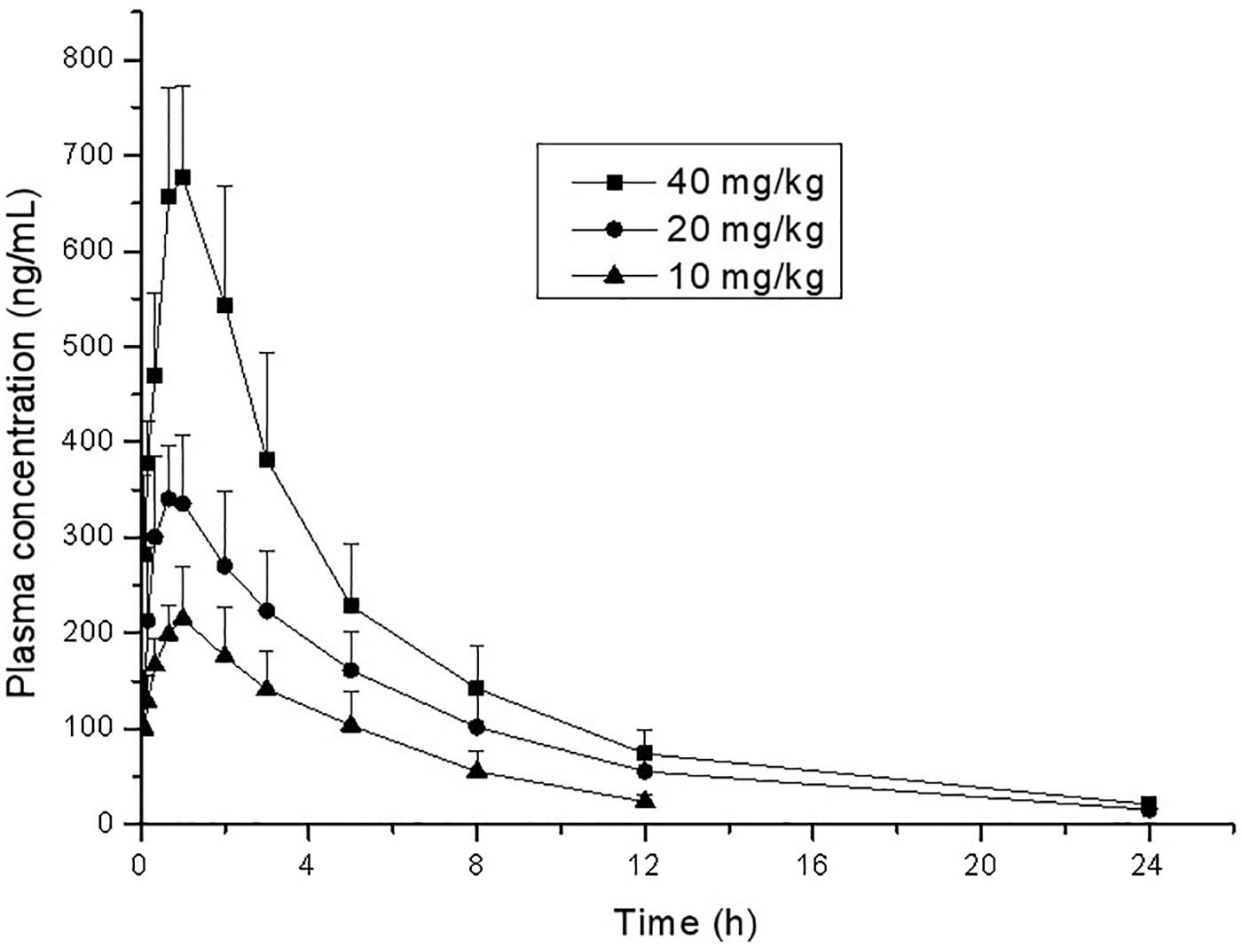
Mean plasma concentrations of thonningianin A after oral administration to rats orally (p.o., 10, 20, and 40 mg/kg).

The assay was applied to the pharmacokinetic study after oral administration of 10, 20, and 40 mg/kg thonningianin A in rats. The mean plasma concentration-time profiles after oral administration are shown in Figure 4. Meanwhile, the mean pharmacokinetic parameters are summarized in Table 4. The results demonstrated that the plasma concentrations of thonningianin A increased rapidly after oral administration of three dosages and reached the mean peak concentrations (*C*_max_) within 0.61–0.83 h, and then the concentrations declined with the elimination time (*t*_1/2_) of 3.69–4.77 h. The AUC_0–t_ of the three group (10, 20, and 40 mg/kg) was 1163.8 ± 301.8, 2304.5 ± 616.0 and 3762.6 ± 727.9 ng h/mL, respectively. AUC_0–∞_ of the corresponding group was 1307.2 ± 328.0, 2407.5 ± 600.4, and 3877.6 ± 786.4 ng h/mL, respectively. Meanwhile, AUC_0–t_/AUC_0–∞_ of the three dosage groups was more than 89.0% (10 mg/kg), 95.7% (20 mg/kg), and 97.0% (40 mg/kg), respectively, far higher than 80%, indicating that the sampling time is long enough to achieve reliable pharmacokinetic parameters of thonningianin A (Chen et al. 2011).

**Table 4.** Mean pharmacokinetic parameters of thonningianin A after oral administration to rats orally.

Pharmacokinetic parameters	Oral administration		
	10 mg/kg	20 mg/kg	40 mg/kg
AUC_0–t_ (ng h/mL)	1163.8 ± 301.8	2304.5 ± 616.0	3762.6 ± 727.9
AUC_0–∞_ (ng h/mL)	1307.2 ± 328.0	2407.5 ± 600.4	3877.6 ± 786.4
MRT_0–t_ (h)	3.94 ± 0.37	5.75 ± 1.08	5.38 ± 0.46
*t*_1/2_ (h)	3.69 ± 0.86	4.90 ± 1.11	4.77 ± 1.14
*C*_max_ (ng/mL)	221.9 ± 47.0	384.9 ± 58.3	713.3 ± 84.3
*T*_max_ (h)	0.83 ± 0.28	0.61 ± 0.33	0.78 ± 0.17
CL (L/h/kg)	8.08 ± 2.10	8.79 ± 2.37	10.7 ± 2.4

## Conclusions

A simple and sensitive LC–MS/MS method was developed for the quantification of thonningianin A in rat plasma. The assay was fully validated for specificity, sensitivity, linearity, precision, accuracy, recovery, carryover, matrix effect and stability in accordance with the US Food and Drug Administration guidelines. The LLOQ of the method was 10 ng/mL. The present method is the first report in terms of the simple precipitation procedure, high sensitivity, and high-throughput efficiency. This validated assay was successfully applied to determine the pharmacokinetic behaviours of thonningianin A in rats.
